# mRNA based vaccines provide broad protection against different SARS-CoV-2 variants of concern

**DOI:** 10.1080/22221751.2022.2081616

**Published:** 2022-06-04

**Authors:** Haomeng Wang, Zhao Chen, Zhenghua Wang, Jin Li, Zhihong Yan, Jinbo Yuan, Airu Zhu, Lan Chen, Ye Liu, Chenlong Hu, Ali Zhu, Guowei Li, Yuehu Li, Jie Deng, Liqiao Ma, Xiuwen Sui, Wei Miao, Junqiang Li, Xiuyu Zheng, Jinhua Piao, Yanfeng Yao, Juhong Rao, Chao Shan, Zhiming Yuan, Jincun Zhao, Tao Zhu

**Affiliations:** a CanSino (Shanghai) Biotechnologies Co., Ltd, Shanghai, People’s Republic of China; b State Key Laboratory of Respiratory Disease, National Clinical Research Center for Respiratory Disease, Guangzhou Institute of Respiratory Health, the First Affiliated Hospital of Guangzhou Medical University, Guangzhou, People’s Republic of China; c CanSino Biologics, Tianjin, People’s Republic of China; d CanSino (Shanghai) Biological Research Co., Ltd, Shanghai, People’s Republic of China; e Institute of Infectious Disease, Guangzhou Eighth People’s Hospital of Guangzhou Medical University, Guangzhou, People’s Republic of China; f Guangzhou Laboratory, Bio-Island, Guangzhou, People’s Republic of China; g Shanghai Institute for Advanced Immunochemical Studies, School of Life Science and Technology, ShanghaiTech University, Shanghai, People’s Republic of China; h Center for Biosafety Mega-Science, Wuhan Institute of Virology, Chinese Academy of Sciences, Wuhan, People’s Republic of China; i State Key Laboratory of Virology, Wuhan Institute of Virology, Chinese Academy of Sciences, Wuhan, People’s Republic of China; j Hubei Jiangxia Laboratory, Wuhan, People’s Republic of China

**Keywords:** COVID-19, mRNA, Beta, Omicron, vaccine

## Abstract

In order to overcome the pandemic of COVID-19, messenger RNA (mRNA)-based vaccine has been extensively researched as a rapid and versatile strategy. Herein, we described the immunogenicity of mRNA-based vaccines for Beta and the most recent Omicron variants. The homologous mRNA-Beta and mRNA-Omicron and heterologous Ad5-nCoV plus mRNA vaccine exhibited high-level cross-reactive neutralization for Beta, original, Delta, and Omicron variants. It indicated that the COVID-19 mRNA vaccines have great potential in the clinical use against different SARS-CoV-2 variants.

## Introduction

During the last two years, the global COVID-19 pandemic has taken millions of deaths all over the world and it still threatens public health. Until now, several drugs have been approved for the treatment of COVID-19 such as antibody drugs, and small molecular inhibitors [1]. Meanwhile, several types of vaccines have been approved for emergency use for the COVID-19 pandemic such as mRNA, viral vector, and recombinant protein-based vaccines [1–6]. Especially, the two approved mRNA vaccines (BNT162b2 and mRNA-1273) indicated that mRNA-based vaccines showed extremely rapidly responding to the viral pandemic [3,4].

As of now, viruses belonging to lineages B.1.1.7 (Alpha), B.1.351 (Beta), B.1.1.28.1 (Gamma), B.1.617.2 (Delta), and B.1.1.529 (Omicron) are classified as variants of concern (VOCs) based on their risk to public health [7]. The Beta, Delta, and the most recent Omicron strains contain several mutations in the spike gene to make these variants show high transmission and immune evasion [5,7–11]. Taking these into concerns, there is still a huge demand of COVID-19 vaccine globally to end the pandemic.

Herein, we presented here the preclinical study of mRNA-based COVID-19 vaccine candidates, mRNA-Beta and mRNA-Omicron. Both the homologous immunization of mRNA vaccines and heterologous immunization of Ad5-nCoV plus mRNA vaccine exhibited high level of cross-neutralizing activity for original, Beta, Delta, and Omicron strains. All of these preclinical results showed that the COVID-19 mRNA vaccines have great potential for the clinical demands against different epidemic SARS-CoV-2 variants.

## Results and discussion

The full-length SARS-CoV-2 spike protein encoded by the sequence-optimized mRNA is based on the original strain, and contains four mutations (K417N, E484K, N501Y, and D614G) found in the Beta variant [7]. The mRNA was obtained through *in vitro* transcription and then encapsulated in LNP through microfluidic system. Main physical characteristics of mRNA-LNP formulations were determined (Table S1) and the *in vitro* expression of antigen encoded mRNA were confirmed in Hep3B cells (Figure S1).

**Figure 1.**
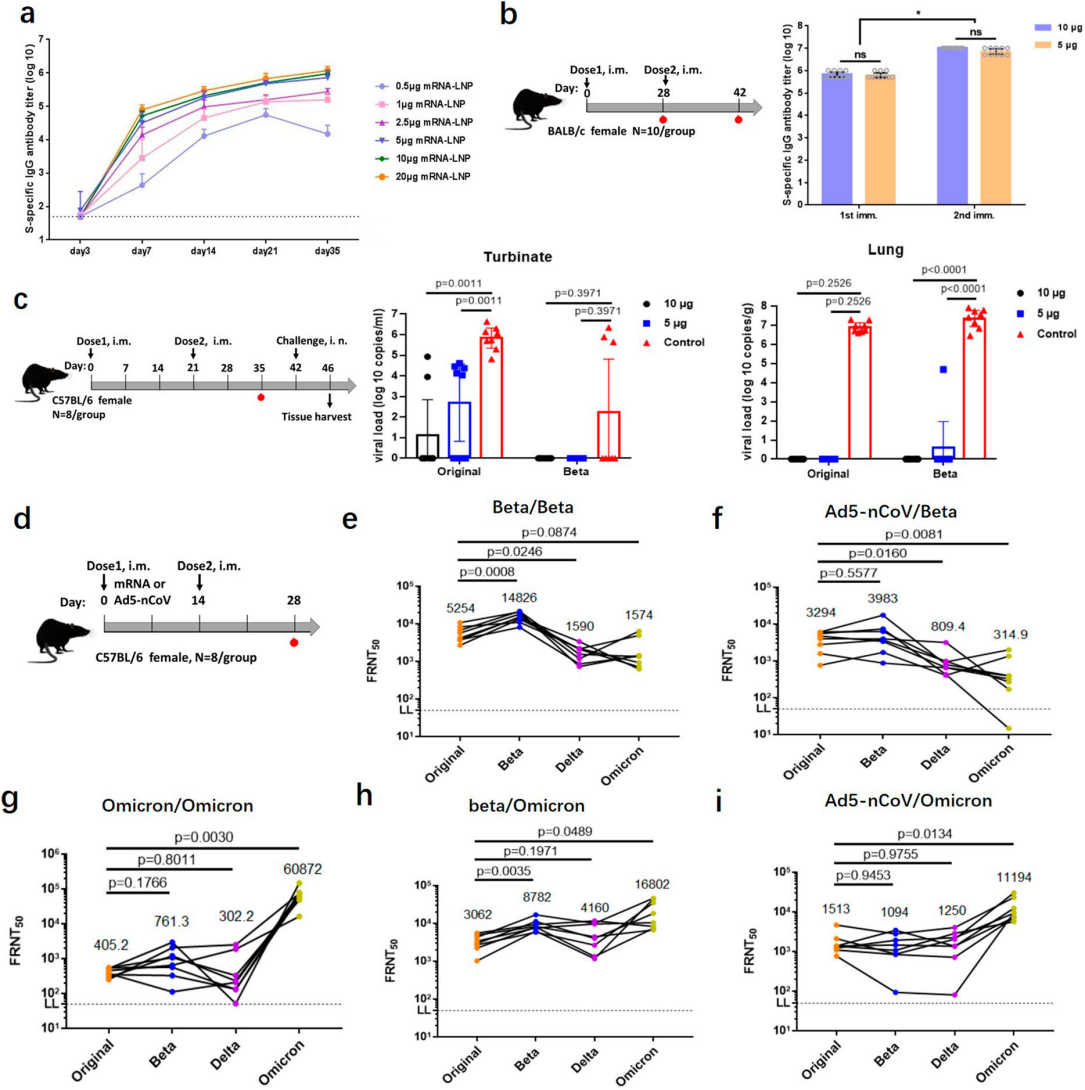
Efficiency of the mRNA-Beta and mRNA-Omicron. (a) Dose-dependent antibody responses of mRNA-Beta. (b) A second dose boost antibody titre. (c) mRNA-Beta attenuated viral replication. (d) Schematic diagram of immunization and sample collection of FRNT assay. (e–f) Cross-reactive neutralization of mRNA-Beta booster plus (e) mRNA-Beta and (f) Ad5-nCoV, (g–i) Cross-reactive neutralization of mRNA-Omicron plus (g) mRNA-Omicron, (h) mRNA-Beta and (i) Ad5-nCoV.

Firstly, dose-dependent antibody responses were determined by immunizing female BALB/c mice with a single dose of 0.5–20 µg mRNA-Beta through intramuscular (i.m.) injection. Detectable S-binding IgG antibodies were induced 7 days post immunization with a dose as low as 0.5 µg and remained at a relatively constant level 35 days post immunization (Figure 1(a)). The S-specific IgG antibody titres after boost shot were significantly higher than that detected pre-boost (Figure 1(b)). 5 µg of mRNA-Beta in mice was sufficient to induce strong antibody responses, and higher doses didn’t further enhance immunogenicity.

To further evaluate *in vivo* protection efficacy of mRNA-Beta in mouse model, hACE2 transgenic mice received two doses of mRNA-Beta at 5 or 10 µg were i.n. challenged with SARS-CoV-2 original and Beta variants. All animals were sacrificed on day 4 post challenge, and lung and turbinate were collected as indicated for the subsequent analysis to determine the degree of virus replication in such organs. SARS-CoV-2 virus replication was efficiently attenuated in both high and low dosage groups compared to the mock-vaccinated group with high virus replication (Figure 1(c)). Viral replication was undetectable in lung. But original virus remains capable of establishing local infection in turbinate. For the Beta variant, no viral replication was detected indicating that the mRNA-Beta vaccine could give effective immune protection in both the upper and lower respiratory tract. It was reported that IgA is thought to be critical for the protection against SARS-CoV-2 viral secretion in upper respiratory tracts. Whether our mRNA-Beta could induce IgA antibody remains to be determined. What is more, mock-vaccinated group developed typical lung lesions, whereas much less symptoms were seen in lung sections of all vaccine-immunized animals (Figure S2). These results demonstrate that two doses of mRNA-Beta vaccination prevent SARS-CoV-2 replication in lung and protect mice from lung lesions.

The cross-variants neutralization capacity to original, Beta, and Delta variants by sera from mice vaccinated twice with mRNA-Beta (5 µg) through a live-virus based FRNT assay were assessed. Additionally, cross-neutralizing capacity for the most recent Omicron variant was also assessed. High neutralization titres against original and Beta variants were elicited by homologous prime-boost vaccination with mRNA-Beta, reaching reciprocal FRNT_50_ geometric mean titres (GMTs) of 5254 (original), 14,826 (Beta), separately (Figure 1(d–e)). These differences may influence the viral replication capability in turbinate (Figure 1(c)). Two doses of mRNA-Beta also gave the same cross-neutralization level against Delta and Omicron with the FRNT_50_ GMTs recorded as 1590 and 1574 which indicated mRNA-Beta could also give relatively protection to Delta and Omicron strains. Furthermore, mRNA-Beta (5 µg) as a booster injection plus heterologous vaccination with the recombinant adenovirus type 5 (Ad5)-vector based COVID-19 vaccine Ad5-nCoV (5 × 10^9^ VP) prime resulted in relatively high levels of neutralizing antibody against the original and Beta variants with FRNT_50_ GMTs recorded as 3294 and 3983, respectively [12]. But it showed comparably lower neutralizing capacity for Omicron variants (Figure 1(f)).

In order to improve the protection efficiency for Omicron variant, we quickly designed and synthesized an Omicron-specific mRNA vaccine (Table S1, Figure S1). Two doses of mRNA-Omicron (5 µg) exhibited a highly specific Omicron variant with the FRNT_50_ reaching 1/60,872. But for original, Beta, and Delta strains, it exhibited low level of cross-reactive neutralization (Figure 1(g)). Compared with mRNA-Beta, the neutralizing antibody elicited by mRNA-Omicron is almost “exclusive” to Omicron strain, which is consistent with other studies [5,13,14]. However, after immunizing with the mRNA-Beta (5 µg) primary, boosting with mRNA-Omicron (5 µg) induced high level of cross-reactive neutralization for all the four variants with the following ranking FRNT_50_ GMTs records: 16,802 (Omicron) > 8782 (Beta) > 4160 (Delta) > 3062 (original) (Figure 1(h)). Compared to mRNA-Beta/mRNA-Beta, the homologous mRNA-Beta/mRNA-Omicron induced less than one fold reduction in GMTs against original and Beta variants, but more than 2.5 fold and 10 fold increased for Delta and Omicron variants, respectively. Meanwhile, Heterologous vaccination of Ad5-nCoV (5 × 10^9^ VP) plus mRNA-Omicron (5 µg) also showed very high-level cross-neutralization for the Omicron variant with the FRNT_50_ value of 1/11,194 and comparable activity to original, Beta and Delta variants (Figure 1(i)). Overall, mRNA-Omicron was suggested as a booster injection in mice vaccinated with primary mRNA-Beta or Ad5-nCoV to improve protection to Omicron variant.

## Conclusion

Overall, our data presented here demonstrated that the mRNA-Beta and mRNA-Omicron could provide broad protection against original, Beta, Delta, and the newly Omicron epidemic SARS-nCoV-2 variants. Two doses of mRNA-Beta could induce broad protection especially for Beta and original variants. Meanwhile, the booster injection of mRNA-Omicron could also improve the protection of primary vaccination of homologous mRNA-Beta or heterologous Ad5-nCoV against the four VOCs, especially for the Omicron variant. All of these data suggested the two mRNA vaccines have great clinical potential to overcome COVID-19 pandemic.

## Supplementary Material

Supplemental Material
