# Supplementary material for: mRNA based vaccines provide broad protection against different SARS-CoV-2 variants of concern
Source: Emerg Microbes Infect. 2022 Jun 4;11(1):1550–3. doi: 10.1080/22221751.2022.2081616 (PMC9176361; doi:10.1080/22221751.2022.2081616)
Supplement: Supplemental Material [file TEMI_A_2081616_SM1731.docx]

**Supplementary materials**

mRNA based vaccines provide broad protection against different SARS-CoV-2 variants of concern

Haomeng Wang^1,3,4,#^, Zhao Chen^2,#^, Zhenghua Wang^1,3,4^, Jin Li^1,3,4^, Zhihong Yan^1,3,4^, Jinbo Yuan^1,3,4^, Airu Zhu^2^, Lan Chen^2^, Ye Liu^1,3,4^, Chenlong Hu^1,3,4^, Ali Zhu^1^, Guowei Li^1,3,4^, Yuehu Li^1,3,4^, Jie Deng^3^, Liqiao Ma^3^, Xiuwen Sui^3^, Wei Miao^3^, Junqiang Li^3^, Xiuyu Zheng^3^, Jinhua Piao^3^, Yanfeng Yao^8^, Juhong Rao^9^, Chao Shan^9,10^, Zhiming Yuan^8^, Jincun Zhao^2,5,6,7,*^ and Tao Zhu^1,3,4,*^

^1^ CanSino (Shanghai) Biotechnologies Co., Ltd, Shanghai 201422, People’s Republic of China

^2^ State Key Laboratory of Respiratory Disease, National Clinical Research Center for Respiratory Disease, Guangzhou Institute of Respiratory Health, the First Affiliated Hospital of Guangzhou Medical University, Guangzhou, Guangdong, 510120, People’s Republic of China

^3^ CanSino Biologics, Tianjin 300457, People’s Republic of China

^4^ CanSino (Shanghai) Biological Research Co., Ltd, Shanghai 201203, People’s Republic of China

^5^ Institute of Infectious Disease, Guangzhou Eighth People's Hospital of Guangzhou Medical University, Guangzhou, Guangdong 510060, People’s Republic of China

^6^ Guangzhou Laboratory, Bio-Island, Guangzhou, Guangdong 510320, People’s Republic of China.

^7^ Shanghai Institute for Advanced Immunochemical Studies, School of Life Science and Technology, ShanghaiTech University, Shanghai, 201210, People’s Republic of China.

^8^ Center for Biosafety Mega-Science, Wuhan Institute of Virology, Chinese Academy of Sciences, Wuhan, Hubei, People’s Republic of China.

^9^ State Key Laboratory of Virology, Wuhan Institute of Virology, Chinese Academy of Sciences, Wuhan, Hubei, 430071, People’s Republic of China

^10^ Hubei Jiangxia Laboratory, Wuhan, Hubei, 430200, People’s Republic of China

^#^ These authors contributed equally

^*^ Corresponding authors: [tao.zhu@cansinotech.com](mailto:tao.zhu@cansinotech.com) (Tao Zhu), [zhaojincun@gird.cn](mailto:zhaojincun@gird.cn) (Jincun Zhao)

**Materials and methods**

**Materials, cell lines and virus**

African Green monkey kidney-derived Vero E6 cells (ATCC, Manassas, VA) s were grown in Dulbecco's modified Eagle's medium (DMEM, GIBCO, Grand Island, NY) supplemented with 10% fetal bovine serum (FBS, GIBCO, Grand Island, NY).

The original strain (GenBank: MT123291), Beta strain (IQTC-IM2122239), Deta strain (IQTC-IM2175251) and Omicron strain (IQTC-Y216017) used for FRNT assay were isolated from COVID-19 patients in Guangzhou. The original strain 2019-nCoV-WIV04 (GISAID: EPI_ISI_402124) and Beta strain (NPRC2.062100001) were used in mice protection study.

**Animals**

Female C57BL/6 mice of 6-8 weeks old were vaccinated intramuscularly twice with a 2-4 week interval. In some experiments, female BALB/c mice of 6-8 weeks old were used. mRNA formulations were diluted in 50 µL of 1 x PBS and mice were inoculated via intramuscular injection into the same hind leg for prime and boost doses. Control mice received PBS. Sample size for animal experiments was determined on the basis of criteria set by the institutional animal care and use committee.

Animal immune serum samples were heated at 56 ℃ for 30 minutes before use. SARS-CoV-2 specific IgG antibody titers were determined by ELISA. Neutralizing antibody titers against SARS-CoV-2 were determined by a live virus-based neutralization as a standard focus reduction neutralization test (FRNT), respectively.

**Preclinical mRNA and lipid nanoparticle production**

The mRNAs contain codon-optimized ORF of S flanked by an optimized capped 5'-capped 5'-UTR and an optimized 3'-UTR followed by a poly(A) tail. Codon and sequence optimization were optimized. The mRNA were produced by IVT (in vitro transcription) of a linear plasmid template using T7 RNA polymerase. The mRNA was purified by removing transcription enzymes, the linear DNA template, and mRNA-related impurities before formulation. LNPs were prepared by mixing a buffer solution of mRNA with an ethanol solution of lipid (DSPC, cholesterol, ionizable lipid and DMG-PEG2000) following microfluidic system. The LNPs were concentrated by tangential flow ultrafiltration.

**LNP characterization.**

The size, polydispersity index and zeta potentials of LNPs were measured using dynamic light scattering (Malvern Panalytical Zetasizer Pro). Diameters are reported as the intensity mean peak average. To calculate the nucleic acid encapsulation efficiency, a modified QuantiT RiboGreen RNA assay (Invitrogen) was used and detected with Synergy LX (BioTek). The electron microscopy of mRNA-LNPs was obtained by using Vitrobot Mark IV (Thermo Fisher Scientific) instrument after depositing on a holey carbon grib. Results of these characterization can be found in Supplementary Table S1.

**In vitro expression of mRNA-LNP**

Hep3B cells were seeded in 24-well plates at 10^5^ cells/well. 24 hours later, the cells were transfected with mRNA-LNP (2.5 µg). Four hours later, the medium was replaced with Opti-MEM. The supernatant was collected at 48 hours after transfection, clarified by centrifugation at 1000 x g, and then mixed with SDS loading buffer. The samples were loaded for SDS-PAGE without heating. The secreted antigen protein was then detected by western blotting with a monoclonal antibody.

**S-specific immunoglobulin ELISA**

SARS-CoV-2 S-specific IgG titers were determined by ELISA assay. Briefly, serial dilutions of inactivated serum were added to blocked 96-well plates coated with recombinant SARS-CoV-2 spike S1 antigen (Sino Biological Inc., 40591-V08H10) and plates were incubated at 37 ℃ for 30 minutes. After three washes with wash buffer, plates were added with HRP-conjugated IgG (ZSGB-Bio) and incubated for 30 minutes at 37 ℃. Plates were then washed with wash buffer and added with chromogen solution followed by 15 minutes of incubation at 37 ℃. The absorbance (450 nm) was read using a microplate reader (BioTek).

**FRNT assay**

Vero cells were seeded in 96-well plates (2x10^4^ cells/well) and incubated until 100% confluent. Serial 10-fold dilutions, starting at 1:20, of serum were prepared in DMEM containing 2% FBS. The diluted sera was then mixed with titerated virus in a 1:1 (v/v) ratio to generate a mixture containing ~ 150-200 FFU/25 µL of viruses, followed by incubation at 37 ℃ for 1 hour. The virus/serum mixture were added to wells of 96-well plates of Vero cell monolayers in duplicate (50 µL/well). The plates were then incubated at 37 ℃ for 1 hour. The mixtures were removed and cells were overlaid with 1.6% CMC (125 µL/well). After further incubation at 37 ℃ for 1 day, the cells were fixed with 4% formaldehyde in PBS (200 µL/well) for 30 min. Following fixation, plates were washed twice with PBS and permeabilization buffer (0.2% Triton, 1% BSA in PBS) was added for 20-30 min. Cells were incubated with an anti-SARS-CoV N primary antibody and then IgG-HRP antibody. Cells were stained with TrueBlue (50 µL/well). Foci numbers were recorded by CTL S6 ultra after rinsing the plates with deionized water. The 50% neutralization titer (FRNT_50_) was calculated by the GraphPad Prism software.

**SARS-CoV-2 mouse challenge**

The SARS-CoV-2 challenge model based on the mouse adapted strain has been characterized in detail. BALB/c mice immunized with two doses of mRNA-Beta were challenged intranasaly with virus (5×10^5^ PFU/mouse) at the indicated times. On day 5 post challenge, all animals were sacrificed, and the lung and turbinate tissues were collected for subsequent viral RNA level determination through RT-qPCR.

**Histopathology assay**

For histopathology, lung tissues from mice were fixed in 4% neutral-buffered formaldehyde for 48 hours, embedded in paraffin, sectioned, and stained with hematoxylin and eosin (H&E). Images were captured using Olympus BX51 microscope.

**Statistical analysis**

All data were analyzed with GraphPad Prism software. No statistical methods were used to predetermine sample size, unless indicated. The investigators were not blinded to allocation during experiments and outcome assessment unless indicated. Unless specified, data are presented as geometric mean ± 95% confidence interval in all experiments. Analysis of variance (ANOVA) was used to determine statistical significance among different groups with geometric means of each group ± 95% confidence interval to calculate significance, depending on the samples, with Tukey’s correction for multiple comparisons. In figure 1b, different dose levels were compared after each immunization by Tukey’s multiple comparisons test and the pre- and post-boost values were compared by unpaired t test with Welch’s correction.

Table S1: Characteristics of mRNA-Beta and mRNA-Omicron formulations

| Vaccine | **mRNA-Beta** | **mRNA-Omicron** |
| --- | --- | --- |
| Diameter | 74.19 nm | 76.93 nm |
| PDI | 0.098 | 0.098 |
| Zeta potential | -1.73 mV | -2.91 mV |
| Encapsulation efficiency | 92% | 96% |
| mRNA integrality | 91% | 88% |
| Electron microscopy | 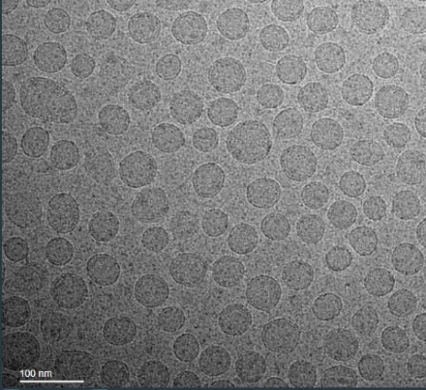 | 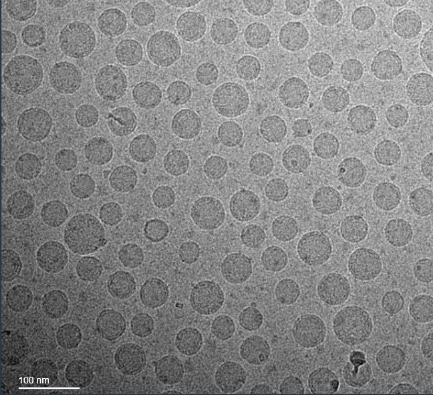 |


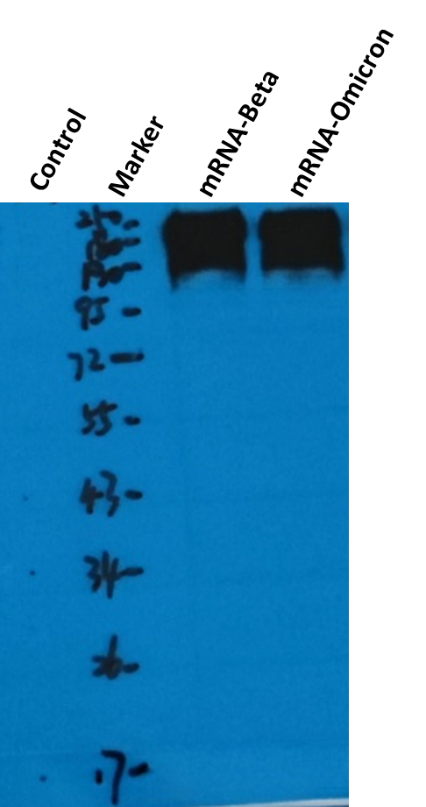


Figure S1: Western blot detection of antigen expression in Hep3B cells while transfected with mRNA-beta and mRNA-Omicron for 24 hours (equal volume saline as negative control).


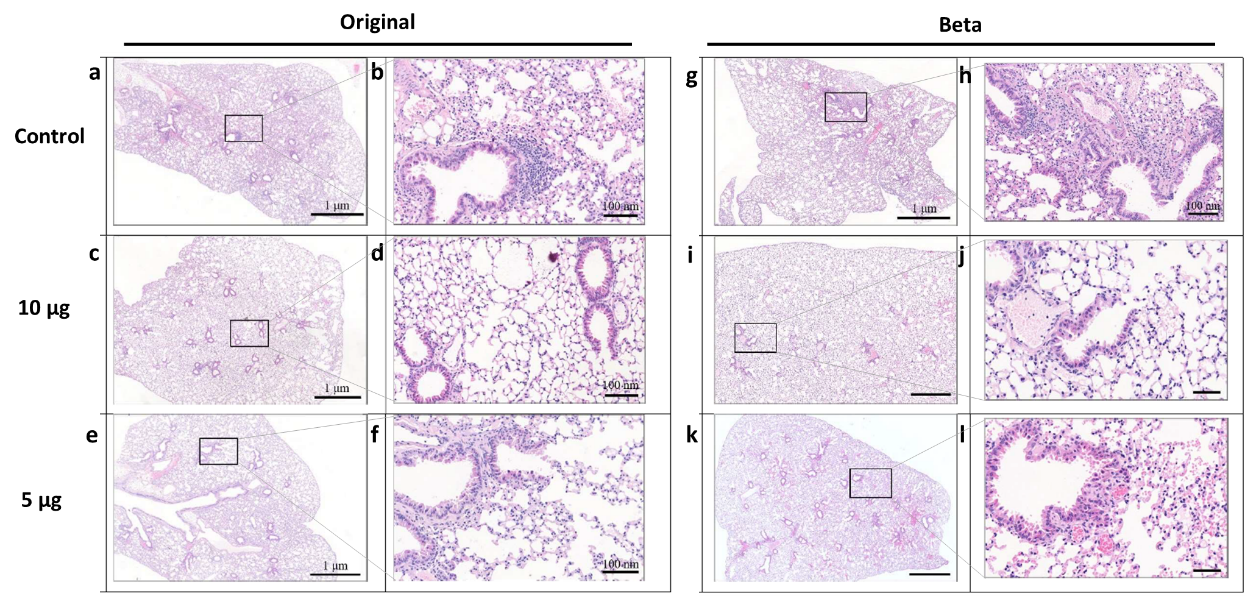


Figure S2: mRNA-beta protects hamster lungs from the pathology caused by SARS-CoV-2 challenge. Lung tissues obtained from the hamsters in Fig. 1c were fixed in formalin and stained with hematoxylin and eosin. Representative micrographs of the hamster lungs, showing extensive acute and mixed inflammatory cell infiltrates in bronchiole and alveoli in a control hamster receiving formulation buffer and paucity of inflammation in a vaccinated animal after challenged with SARS-CoV-2.
